# Very-Long-Chain Resorcinolic Lipids of *Ailanthus altissima* Samaras

**DOI:** 10.3390/molecules30142970

**Published:** 2025-07-15

**Authors:** Elżbieta G. Magnucka, Robert Zarnowski, Przemysław Bąbelewski

**Affiliations:** 1 Laboratory of Biogeochemistry and Environmental Microbiology, Department of Plant Protection, Wrocław University of Environmental and Life Sciences, Grunwaldzka St. 53, 50-357 Wrocław, Poland; 2 Department of Medicine, School of Medicine and Public Health, University of Wisconsin, 5225 Microbial Sciences Building, 1550 Linden Dr., Madison, WI 53706, USA; rzarnowski@wisc.edu; 3 Department of Horticulture, Wrocław University of Environmental and Life Sciences, Grunwaldzki Sq. 24a, 50-363 Wrocław, Poland; przemyslaw.babelewski@upwr.edu.pl

**Keywords:** ailanthus samaras, antifungal activity, 5-*n*-hentriacontylbenzene-1,3-diol, 5-*n*-nonacosylbenzene-1,3-diol, *Fusarium*, *Rhizoctonia*, phenolic lipids

## Abstract

Two new very-long-chain 5-*n*-alkylresorcinol (AR) homologues, that is, 5-*n*-nonacosylbenzene-1,3-diol and 5-*n*-hentriacontylbenzene-1,3-diol, were isolated from acetone extracts of *Ailanthus altissima* samaras. These phenolic compounds were detected in nearly equal proportions, although their total content varied considerably between samples from urban-grown trees. No correlation was observed between AR levels and the physiological state of the tree, suggesting that environmental conditions may strongly influence AR biosynthesis in *A. altissima*. Furthermore, the isolated AR mixture exhibited antifungal activity against soil-borne phytopathogens of the genera *Fusarium* and *Rhizoctonia*.

## 1. Introduction

*Ailanthus altissima* (Mill.) Swingle (family Simaroubaceae), commonly known as ailanthus, a tree of heaven, Chinese sumac, or stinking sumac, is an extremely successful invasive tree species frequently found in urban areas. This exotic ornamental plant is native to Taiwan, as well as northeast and central China. However, today it is distributed throughout the world, with the exception of Antarctica [1,2,3], and is considered a problematic species due to its negative impact on native plant populations.

Ailanthus reproduces rapidly both sexually (via seeds) and asexually (through vegetative sprouts). Seeds are produced by female trees in late summer to early fall, enclosed within flat, twisted, winged, papery, tan- to pink-colored fruit structures called samaras [4].

Extracts of *A. altissima* are widely used in traditional Asian folk medicine to treat a range of disorders, including ascariasis, asthma, bleeding, colds, epilepsy, gastrointestinal disorders, and ophthalmic diseases [5]. Several studies have also shown that ailanthus is a promising source of antiviral [6], antitumor [7,8,9], antiplasmodial [10], insecticidal [11], and antimicrobial agents [12]. Some of these compounds can be successfully used as efficient phytotoxins in weed management [13]. These biological activities are attributable to the presence of various natural phytochemicals, including alkaloids, coumarins, flavonoids, lignans, lipids, phenols, quassinoids, sterols, and triterpenoids [14]. More than 200 components have been identified to date in the leaves, fruits, and bark of the roots, stems, or trunk of ailanthus [10,14,15]. However, among the numerous compounds identified in the fruit of this tree [6,16,17], the presence of resorcinol lipids has not yet been demonstrated.

Resorcinolic lipids, also called 5-*n*-alk(en)ylresorcinols (ARs), are long-chain homologues with odd numbers (typically 15–25 carbons) of orcinol (1,3-dihydroxy-5-methylbenzene). These secondary amphiphilic plant metabolites originating from the polyketide metabolic pathway exhibit potent antimicrobial activity [18]. Their activity against soil-borne phytopathogenic fungi often earns them the designation of natural biofungicides. To date, their antifungal activity has been demonstrated primarily against ascomycetous pathogens of the genera *Aspergillus*, *Penicillium*, and *Fusarium* [18,19,20]. Interestingly, this activity of these phenolic compounds is related to their structures [20,21].

This study reports on the isolation and characterization of new very-long-chain 5-*n*-alkylresorcinols from *A. altissima* samaras. Furthermore, the antifungal activity of the newly isolated phenolic mixture was evaluated against selected phytopathogens of the genera *Fusarium* and *Rhizoctonia*.

## 2. Results and Discussion

**Figure 1:**
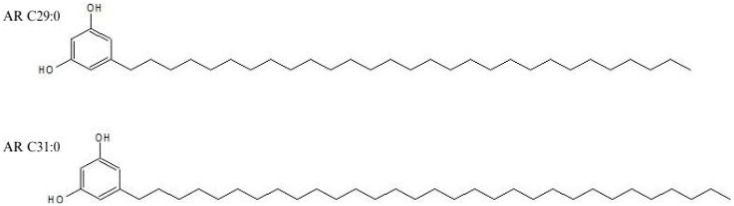
Structures of resorcinolic compounds identified in *A. altissima* samaras.

This study reports on novel very-long-chain alkylresorcinol homologues in *A. altissima* samaras (Figure 1).

The isolation procedure used here is commonly applied to obtain the pure resorcinolic lipid fractions, mainly from various plant materials [22]. Acetone extracts were separated on a set of TLC silica gel plates, and alkylresorcinol bands were visualized by staining with the diazonic salt Fast Blue B [23]. These bands exhibited characteristic reddish-violet color due to the effect of the coupling of this diazo dye with alkylresorcinol particles and R_f_ values identical to those of authentic 1,3-dihydroxy-5-alkylresorcinols [18]. Fast Blue B-positive bands were scraped off the plates and further separated on C18 RP-TLC plates, which yielded two reddish-violet bands strongly suggesting the presence of unusual alkylresorcinols substituted with very long side chains. Subsequent TLC analysis on silver-impregnated silica gel plates revealed the presence of resorcinolic homologues saturated solely [22].

**Figure 2:**
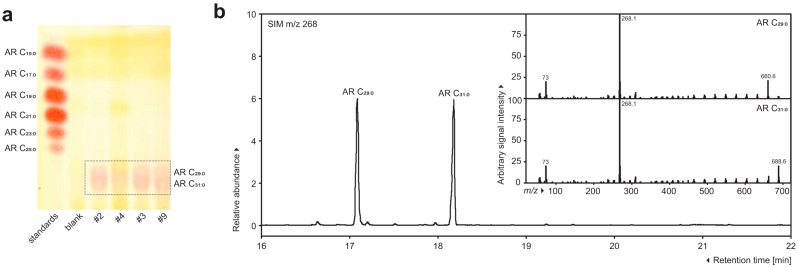
Very-long-chain 5-*n*-alkylresorcinols are present in *Ailanthus altissima* samaras. (**a**) Thin-layer chromatography separation on C18 RP-TLC plates yielded two reddish-violet bands strongly suggesting the presence of unusual alkylresorcinols substituted with very long side chains. Lines #2, #4, #3, and #9 show exemplary samples. (**b**) Gas chromatography analysis profiling revealed the presence of two alkylresorcinol homologues substituted with long side chains consisting of 29 and 31 carbon atoms. The selected ion monitoring (SIM) chromatogram at *m*/*z* 268 revealed distinct peaks at approximately 17.1 and 18.2 min. The inset shows mass spectra of the newly identified alkylresorcinol derivatives.

The structure of the isolated compounds was elucidated using a combination of chromatographic and spectroscopic methods. The UV spectra with two close peaks at λmax 278 and 282 nm did not show significant differences from those of the standards. The GC/EI-MS analysis showed the presence of two major alkylresorcinol homologues in the sample, identified as C29:0 and C31:0 based on their retention times and mass spectrometric fragmentation patterns. The selected ion monitoring (SIM) chromatogram at *m*/*z* 268 revealed distinct peaks at approximately 17.1 and 18.2 min, corresponding to the molecular ions of the respective alkylresorcinol derivatives. The electron ionization mass spectra of both phenolic compounds exhibited characteristic fragmentation patterns with base peaks at *m*/*z* 268, confirming their molecular weights. Additional prominent fragments were observed at *m*/*z* 73, typical of alkylresorcinol structures, and at *m*/*z* 660.6 and 688.6. The chromatographic separation demonstrated excellent resolution between the two homologues, with the C29:0 derivative eluting before the C31:0 homologue, consistent with the expected elution order based on carbon chain length (Figure 2).

In the ten seed samples examined, 5-*n*-hentriacontylbenzene-1,3-diol-C31:0 was newly identified, together with the already known 5-*n*-nonacosylbenzene-1,3-diol-C29:0. The latter was found in the seeds of *Elymus repens* (L.) Gould, *Bromus mollis* L., and *Leymus arenarius* (L.) Hochst. [24].

The relative homologue composition and the total amounts of alkylresorcinols in *A. altissima* samaras are presented in Table 1.

The content of resorcinolic lipids in the samaras varied from 13.2 to 33.9 mg kg^−1^ and slight variations in homologue composition were observed between particular samples. Both detected resorcinol homologues were present in almost equal amounts, and only minor alterations in their contents were determined. We determined that 5-*n*-nonacosyl-1,3-dixydroxybenzene constituted 42.5% to 50.1% of the total resorcinol pool in *A. altissima* samaras, while the content of 5-*n*-hentriacotyl-1,3-dihydroxybenzene was slightly higher and ranged from 49.9% to 57.5%. The observed divergences in the resorcinol content and composition between individual trees of *A. altissima* did not correlate with the diameter of the tree trunk (which is directly related to the age of the tree). Therefore, we conclude that these differences resulted from the natural variability determined by broad-comprehended, so far undefined, environmental parameters directly relevant to the biosynthesis of resorcinolic lipids in these tree plants. The literature indicates that the level of these phenolic compounds, mainly in cereals, can be modulated by various factors such as light, temperature, microbial infection, and pesticides [18,21,25].

The presence of resorcinolic lipids has been described in several plant species belonging to the Anacardiaceae, Araceae, Ginkgoaceae, Myristicaceae, Proteaceae, Primulaceae [18], and Fabaceae families [22,26]. Our results indicate that *A. altissima* samaras is a new natural source of long-chain alkylresorcinols. Furthermore, this finding indicates that resorcinolic lipids might be more abundant in the plant kingdom. Alkylresorcinols, like those described in this work, exhibit some biological activities [18]. Among these activities, resorcinolic lipids exhibit antibacterial and antifungal activities [19,20,27]. For this reason, the second objective of this work was to estimate the antifungal activity of ARs extracted from *A. altissima* samaras. The results obtained are summarized in Table 2.

The calculated IC_50_ values (the concentration of compounds required to inhibit fungal growth by 50%) showed significant antifungal activity from the mixture of these two homologues against the phytopathogenic fungi tested: *F. culmorum*, *F. oxysporum*, *R. cerealis*, and *R. solani*. As previously reported on rye AR [19], *Fusarium* spp. was more resistant than two other *Rhizoctonia* species tested. Analysis of the fungistatic potentials of the 5-*n*-alkylresorcinol extract of durum wheat grains against various species of *Fusarium* also confirmed this observation [27]. The activity of the new AR homologues of *A. altissima* expressed by IC_50_ was in the range of 64.4–178.6 µg mL^−1^ for *F. culmorum* and *F. oxysporum*, respectively, while the sensitivity levels of the tested isolates of *Rhizoctonia* spp. were comparable, but reduced and varied from 12.8 to average 14.4 µg mL^−1^ for *R. cerealis* and both strains of *R. solani*. The values obtained for *Rhizoctonia* fungi were markedly lower than those presented in previous studies for the shorter carbon-chain saturated compounds. Magnucka et al. [20], for example, showed that homologues C21:0 and C23:0 at a dose of 50 µg mL^−1^ inhibited the radial growth of *Rhizoctonia solani* F93 mycelium on average by 42.05% and 44.06%, respectively. Therefore, the antimicrobial activity of these phenolic lipids appears to be higher in those with longer carbon chains. This fact is consistent with earlier results presented by Zarnowski et al. [28], who showed that the reduction in mycelial growth of *Rhizoctonia solani* by saturated homologues (C15:0, C19:0 and C23:0) rose as their side-chain length increased. Furthermore, this correlation characterizes various other natural and synthetic compounds with hydrophobic alkyl chains, for example, fatty acids and their derivatives [29,30] or ionic liquids [31]. In summary, the discovery of very-long-chain alkylresorcinols in *A. altissima* samaras may support the thesis of the protective role of these phenolic compounds in the seed biology of this plant.

## 3. Materials and Methods

### 3.1. Chemicals and Materials

Chemicals including acetone, chloroform, ethanol, ethyl acetate, diethyl ether, formic acid, hexane, and methanol were supplied by Avantor Performance Materials Poland S.A. (Gliwice, Poland). Pure 5-*n*-pentadecylresorcinol (C15:0), Fast Blue B salt (DBB), and Potato Glucose Agar (PGA) were sourced from Merck KGaA (Darmstadt, Germany). Standards of authentic resorcinol homologues (C15:0–C25:0) were isolated from rye kernels as previously described [32]. All TLC plates utilized in this study were provided by Merck KGaA (Darmstadt, Germany).

### 3.2. Plant Material

The samaras of *A. altissima* used in this study were collected from trees that were growing in ten different locations in Wroclaw, Poland. The samaras, after drying at 50 °C for 48 h, were stored in moisture resistant containers at room temperature. Each sample was analyzed in triplicate.

### 3.3. Isolation and Purification of Resorcinolic Lipids

Resorcinolic lipid fractions were isolated from dried samaras using the modified method of Zarnowski and Kozubek [22]. The sample was extracted three times with acetone (24 h each), and the combined filtrates were evaporated under reduced pressure. The residue was dissolved in 0.2 mL of ethyl acetate and applied to a Si60 TLC plate. Separation was performed first with chloroform and then with chloroform/ethyl acetate (85:15, *v*/*v*). After solvent evaporation, both edges of the plate were sprayed with 0.05% (*w*/*v*) aqueous DBB. The target band was scraped, transferred to a 1 × 10 cm column, and eluted with 120 mL of ethyl acetate. The eluates were pooled and dried under reduced pressure. The extract was redissolved in 0.2 mL of ethyl acetate and subjected to TLC with hexane/diethyl ether/formic acid (70:30:1, *v*/*v*). The purification steps were repeated and the final alkylresorcinol fraction was dissolved in 0.2 mL of chloroform.

### 3.4. Quantitative Determination of Resorcinolic Lipids

Alkylresorcinol content was determined using the colorimetric method of Gajda et al. [23]. A calibration curve (1–10 μg, R^2^ = 0.983) with 5-n-pentadecylresorcinol (C15:0) as standard was prepared. Measurements were performed in triplicate.

### 3.5. Identification and Determination of Alkylresorcinol Homologue Compositions

To determine the composition of the homologues according to the length of the side chain, reverse-phase thin-layer chromatography was performed on RP18 HPTLC plates [33]. For the analysis of the presence and composition of homologues according to the unsaturation of the side chain, the argentation chromatography technique on silver-impregnated silica gel was used [22]. Chromatograms were developed in pure methanol and stained with an aqueous 0.05% Fast Blue B solution.

UV spectra were measured with an ethanol solution on a Cintra 20 spectrophotometer (GBC Scientific Equipment Ltd., Melbourne, Victoria, Australia). The homologue composition were determined using gas chromatography coupled with mass spectrometry (GC/EI-MS) by the method described by Magnucka et al. [25], with some minor method adjustments. TMS-derivatized alkylresorcinols (1 µL) were injected into an HP 5890 Series II gas chromatograph (Hewlett-Packard, Palo Alto, CA, USA) couple with a JEOL SX-100 Mass Spectrometer mass spectrometer (Japan Electron Optics Laboratory Co., Ltd., Tokyo, Japan) at 70 eV with a helium flowrate of 1 mL min^−1^. Samples were separated on a DB-1 column (∅ 0.25 mm diameter × 15 m length, 0.25 µm film thickness; G&L Science, Tokyo, Japan). The sample injection port temperature was set at 250 °C. The column oven temperature was programmed as follows: 80 °C for 1 min, 30 °C/min to 230 °C, 10 °C/min up to 320 °C and 320 °C for 7 min. The identification of two alkylresorcinol homologues was obtained from the molecular ion and common base peak at *m*/*z* 268, which is characteristic of those molecules. The retention time of each saturated homologue was 17.1 min (M^+^ 660.6, C29:0) and 18.2 min (M^+^ 688.6, C31:0).

### 3.6. Antifungal Activity of Alkylresorcinols

The inhibitory activity against phytopathogenic fungi, *Fusarium culmorum* (Wm. G. Sm.) Sacc. F1, *Fusarium oxysporum* Schltdl. R1, *Rhizoctonia cerealis* E.P. Hoeven F71, *Rhizoctonia solani* J.G. Kühn F92 and F93, was determined using the medium poisoning method [19]. The fungal isolates were incubated on potato dextrose agar (PDA) in which ARs of *A. altissima* samaras were added at concentrations of up to 0.4 mg mL^−1^. In this analysis, a mixture of these two homologues obtained according to the procedure described above from one selected sample with a high initial biomass of dried samaras was used. The control medium contained only an organic solvent. The plates were thoroughly evaporated in a laminar box. Then, 5 mm-diameter discs of 7-day-old cultures of the respective fungi were placed in the center of the Petri dishes. Each dose was analyzed in triplicate and radial growth was monitored for five consecutive days. The IC_50_ values, expressed in µg mL^−1^, were calculated using Prism 8 (GraphPad Software Inc., San Diego, CA, USA).

### 3.7. Statistical Analysis

The results of the conducted analyses are presented as the mean of three independent experiments. Standard deviations were calculated for all results obtained. Antifungal activity data and total alkylresorcinol levels were subjected to Tukey’s HSD (Honestly Significant Difference) post hoc test (*p* ≤ 0.05). Statistical analysis was performed using R version 4.4.2.(R Core Team, Vienna, Austria).

## 4. Conclusions

This study presents the first identification of two very-long-chain alkylresorcinols, C29:0 and C31:0, in *Ailanthus altissima* samaras. These phenolic compounds were detected in all the samples analyzed, although their total concentrations varied independently of tree diameter. Furthermore, the mixture of these homologues exhibited notable antifungal activity, particularly against *Rhizoctonia* spp., suggesting a role of these compounds in the defense of seeds against some phytopathogens.

## Figures and Tables

**Table 1:** Alkylresorcinols of *Ailanthus altissima* samaras.

Sample No.	Trunk Diameter ^b^ [cm]	Resorcinol Content ^a^ [mg kg^−1^ DW]	Homologue Composition [%]	
			C29:0	C31:0
1.	142	13.2 ^c^	50.1	49.9
2.	140 and 133 ^d^	14.8 ^c^	48.6	51.4
3.	93	16.9 ^c^	48.6	51.4
4.	70	21.1 ^b^	42.5	57.5
5.	260	22.7 ^b^	50.1	49.9
6.	90 ^c^	25.6 ^b^	44.0	56.0
7.	127	28.8 ^ab^	44.8	55.2
8.	110	31.1 ^a^	42.8	57.2
9.	239	33.0 ^a^	46.5	53.5
10.	164	33.9 ^a^	49.0	51.0

^a^ Mean of three replications from three independent samples. The standard deviation for homologues did not exceed 2%. Values that share any letter are not significantly different at *p* ≤ 0.05 (Tukey HSD). ^b^ Measured 130 cm above ground level. ^c^ Withered in 30%. ^d^ Two-trunk tree.

**Table 2:** Antifungal activity of 5-*n*-alkylresorcinols of *A. altissima* versus soil-borne phytopathogens.

**IC_50_** **[µg mL^−1^]**	***Fusarium***		***Rhizoctonia***		
	*culmorum* F1	*oxysporum* R1	*cerealis* F71	*solani* F92	*solani* F93
	64.4 ± 3.6 b	178.6 ± 14.8 a	12.8 ± 1.1 c	14.6 ± 1.7 c	14.2 ± 1.7 c

Data are shown as mean ± standard deviation; means that share any letter are not significantly different at *p* ≤ 0.05 (Tukey HSD).

## Data Availability

Data are available on request.
